# Design of the BiRmingham Early Detection In untREated psyChosis Trial (REDIRECT): cluster randomised controlled trial of general practitioner education in detection of first episode psychosis [ISRCTN87898421]

**DOI:** 10.1186/1472-6963-5-19

**Published:** 2005-03-08

**Authors:** Lynda Tait, Helen Lester, Max Birchwood, Nick Freemantle, Sue Wilson

**Affiliations:** 1 Department of Primary Care and General Practice, The University of Birmingham, UK; 2 School of Psychology, The University of Birmingham, UK

## Abstract

**Background:**

Treatment delay in first episode psychosis is common. As general practitioners are the first point of contact for many individuals with first episode psychosis, they are well placed to detect the early symptoms and make urgent referrals to specialist secondary care services. However, early psychosis is often difficult to detect. The primary objective of the Redirect trial is to estimate whether an educational intervention targeted at general practitioners increases the general practitioner referral rate of young people with first episode psychosis to Early Intervention Services.

**Methods/design:**

This paper describes the design of a stratified-cluster randomised controlled trial of an educational intervention on first episode psychosis in primary care. The primary outcome is the number of general practitioner referrals of young people with first episode psychosis to Early Intervention Services. Secondary outcomes are duration of untreated psychosis, time to recovery, use of the Mental Health Act, and general practitioner consultation rate. Young people with first episode psychosis referred to Early Intervention Services will be recruited over a two-year period from 1 March 2004. Seventy-eight out of 89 eligible general practices were recruited. The educational intervention has been implemented and evaluated by general practitioners. The education was well received and considered relevant to clinical practice by the general practitioners.

**Discussion:**

The results suggest that the recruitment strategy and implementation of the educational intervention are feasible and acceptable in a primary care setting. The Redirect trial will provide robust information about the efficacy of an evidence-based complex educational intervention targeted at general practitioners on referral rates of young people with first episode psychosis to Early Intervention Services.

## Background

Studies have shown that young people with first episode psychosis (FEP) experience lengthy delays between the onset of psychotic symptoms and receipt of treatment [1,2]. The average period from first onset of psychosis to initiation of adequate treatment (duration of untreated psychosis, or DUP) is one to two years [3]. During this untreated period, irreversible biological, social and psychological damage may take place [2], and a delay in treatment is associated with poorer short-term outcome and slower recovery [1]. Early intervention in this 'critical period' [4] is therefore important for both patients and families. Strategies to reduce DUP include providing early access to specialist mental health services, such as early intervention services (EIS), and improving recognition of FEP by educating primary care professionals [5].

The integration of mental health and primary care services around the world, in developed as well as developing countries, has been widely advocated [6-10]. As part of this process, a range of national and international policy developments have occurred, with the aim of improving the identification and management of mental illness in primary care [11-18]. In the United Kingdom (UK), for example, standards two and three of the Mental Health National Service Framework [16] require primary care to provide effective identification, assessment, and treatment of people with mental illness, including appropriate early referral to specialist services. National guidance on schizophrenia [19] and the inclusion of mental health indicators for care of people with serious mental illness in the new GP contract [20] further strengthen the role and responsibilities of primary care by encouraging a more systematic approach to care, including the use of protocols and referral guidelines.

As part of the UK policy response to improve the early detection and treatment of FEP, in 2000, the NHS Plan prioritised the development of 50 "early intervention" teams across England and Wales to provide specialist mental health services for all young people aged 14–35 with a FEP [21]. General practitioners (GPs) are well placed to play a greater role in the identification and management of FEP, as they are usually the first point of patient contact [22], and GP involvement in the management of psychosis is associated with reduced use of the Mental Health Act[23]. However, primary care health professionals cannot refer to EIS without knowledge of FEP and an understanding of the EIS referral system.

The limited evidence base in this area has, however, consistently shown a need for improvement in the detection and management of FEP in primary care. Early detection is a challenge for many GPs, since psychosis does not present in "neat packages" and can take several months to emerge [24]. The prodrome largely consists of non-specific psychological and social disturbances of varying intensity, which is possibly why GPs experience difficulty in distinguishing FEP from normal adolescent behaviour [25,26]. GPs also experience uncertainty about how to identify FEP, treat FEP appropriately, and access specialist mental health services [25,26]. These data suggest the importance of training GPs to improve early detection and management of FEP to reduce the delay between onset of symptoms and initiation of treatment.

Recent reports indicate that promoting early detection in a community based setting can be successful [27,28]. A Scandinavian community education programme in the identification of FEP, for example, led to reduction in DUP from 1.5 years (mean) to 0.5 years [27,29]. However, the Redirect trial is the first randomised controlled trial aimed at educating GPs about FEP.

The Redirect study team designed an evidence based "complex" educational intervention that addressed the knowledge, skills, and attitudes of GPs about FEP. A refresher educational intervention was planned and implemented to reinforce knowledge and skills acquired in the initial educational intervention and to promote positive attitudes towards young people with FEP, given that most GPs see only one or two new people with FEP each year. The intervention is being used in a stratified-cluster randomised controlled trial to evaluate the effect of the educational intervention on GP referral rates of young people with FEP to EIS.

### Study aims

The primary aim of the Redirect trial is to estimate whether an educational intervention targeted at GPs increases the GP referral rate of young people with FEP to EIS.

## Methods/design

### Setting, eligibility and recruitment of practices

Ethics Committee approval was obtained from Sandwell & West Birmingham, South Birmingham and East Birmingham Research Ethics Committees. The Redirect trial was conducted in three Primary Care Trusts (PCTs) within Birmingham in the UK, which encompass the areas of Aston, Handsworth, Ladywood, Nechells, Sandwell, Small Heath, Sparkbrook, Sparkhill, Soho, and Washwood Heath. Eighty-nine general practices within these three PCTs with the authority to refer to EIS were eligible for inclusion to the trial. These included 74 general practices located within the catchment area of the Heart of Birmingham Teaching PCT (HoB tPCT), 14 practices within the catchment area of Eastern Birmingham PCT, and one practice within the catchment area of South Birmingham PCT.

Practice recruitment was facilitated by a letter to each practice from the study team and from the Chief Executive of the HoB tPCT, presentations by the second author at the HoB tPCT protected "learning time" sessions (where the PCT enabled GPs to close their practices and attend by paying for locum doctor cover) and an evening meeting attended by the practices within the catchment area of Eastern Birmingham PCT. Practices received a quarterly newsletter post randomisation during the study period. The newsletter was designed to encourage continued interest and participation in the Redirect trial.

Consecutive patients with FEP who are referred by the Redirect trial GPs to the two Birmingham EIS over a two-year period from 1 March 2004 to March 2006 are eligible for inclusion in the study. Patients must also be aged between 14 to 30 years, in line with the Mental Health Policy implementation guide for Early Intervention [30], and have an ICD-10 [31] chart diagnosis of schizophrenia or related disorders (F20, F22, F23, F25, F28, F31). The exclusion criteria are patients with a primary diagnosis of substance use disorder, mood disorder, or organic mental disorder, current criminal proceedings, serious concurrent physical illness, institutional residence, learning disability, or inability to provide informed consent (see Table 1).

**Table 1:** Patient inclusion and exclusion criteria

*Inclusion criteria*

• First episode psychosis
• Aged 14–30 years
• (ICD-10: F20, 22, 23, 25, 28, 31)
• Living within the catchment area

*Exclusion criteria*

Patients with a history of prior psychosis in receipt of antipsychotic medication

Patients with a primary diagnosis of substance use disorder or organic disorder

Institutional residence

Patients with learning disability

Inability to provide informed consent

Patients with current criminal proceedings

Patients with serious concurrent physical illness

### Design and randomisation of general practices

**Figure 1:**
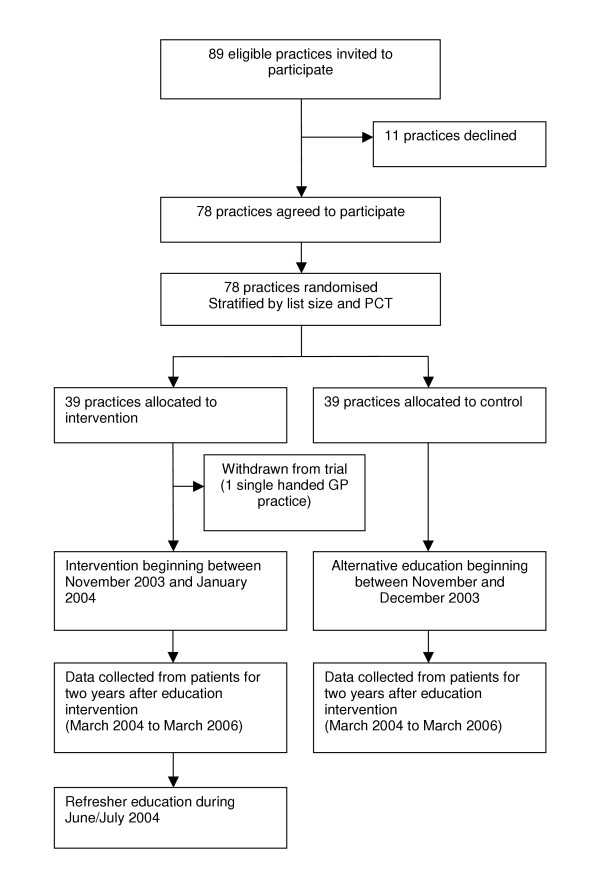
GP enrolment, randomisation and flow of practices through trial

The study is a stratified-cluster randomised controlled trial design [32,33]. The cluster design at practice level reduces the risk that the educational intervention delivered to GPs will be contaminated by interaction between GPs within a single practice. Practices were randomly allocated after stratifying by list size (more or less than 3,500 patients) and PCT (HoB or not). These strata were used to ensure balance on the number of practices where no referrals of FEP might occur and to account for the differential prevalence of FEP across the study area. Practices were randomly allocated to either the intervention (39 practices) or control group (39 practices) by NF, who was blind to practice identity, using computer generated random numbers. Figure 1 shows the trial profile summarising practice recruitment and retention.

### The educational intervention

General practices were randomised to receive either the educational intervention on detecting FEP (intervention practices) or to an alternative educational session on cognitive behaviour therapy for depression (control practices) to control for any possible "attention" effect. In developing the educational intervention, the study team followed the five phases of the framework for the design and evaluation of complex health interventions proposed by the Medical Research Council and Campbell et al[34,35], and incorporated evidence from systematic reviews and guidelines on changing professional practice [36,37]. A brief 17-minute video depicting GP consultations with young people with FEP was shown to all members of the intervention practices, and the trial educators (first and second authors) then led a 15-minute question and answer session. The video addressed GP attitudes and negative stereotypes of people with FEP and also included elements of didactic factual teaching on, for example, links between drug use and psychosis, key warning symptoms of psychosis, including recognition of negative symptoms, and how to ask questions about sensitive issues such as hearing voices and suicidal ideas. A booklet summarising the content of the video and a two-sided laminated 'tip' sheet on FEP symptoms and useful questions to ask patients were also given to all intervention group GPs.

All intervention GPs viewed the video between November 2003 and January 2004. Twenty-one general practices (54%) viewed the video during 'protected learning' time and 18 general practices (46%) viewed the video at their premises during their own time. At the end of the education session, GPs were asked to evaluate three specific elements of the video on a five-point rating scale: key information on FEP, useful questions to ask during consultations, and personal confidence in detecting FEP. GPs were also asked what they found most and least useful about the video.

Refresher educational sessions for intervention practices were conducted in small groups, with training events spread over three days from 29 June 2004 to 1 July 2004. The refresher training consisted of personal testimonies from two service users, a service user personal testimony presented in a brief 10 minute video, and a presentation from an EIS representative on the EIS acceptance criteria and referral process. GPs were again asked to provide feedback on specific components of the training using a similar five-point rating scale.

### Outcomes

The primary outcome will be measured at practice level (unit of randomisation) by assessing the difference in the number of referrals of young people with FEP to secondary care services between study groups. Secondary outcomes are DUP, time to recovery, use of the Mental Health Act (at patient level), and GP consultation rate (at practice level). Instruments used in the evaluation are summarised in Table 2.

**Table 2:** Summary of research assessments

Instrument	Baseline	Follow-up
SCAN [38]	x	
PANSS [41]	x	x
Insight Scale [39]	x	x
Early Signs Scale [40]	x	x
DUP [42]		x
PAS [44]		x
Encounter Form [45]		x

### Baseline assessments and follow-up

The two field researchers are approaching all new FEP referrals to the two EIS in inner-city Birmingham to seek informed consent and entry to the trial. Patients meeting eligibility criteria and providing written informed consent are interviewed by the research team at baseline and at a four-month follow-up interview. At baseline, data are collected on socio-demographic factors and psychosis symptoms during the past month using the Schedules for Clinical Assessment in Neuropsychiatry (SCAN) version 2.0 [38]. The SCAN is a set of instruments used to assess, measure, and classify psychopathology associated with adult major psychiatric disorders. Part two, which includes psychosis disorders, was felt most applicable for use in this study.

### Insight

Insight is measured at baseline and at four months using the Insight Scale [39], an eight-item self-report scale designed to be sensitive to changes in levels of insight. The scale captures each of three widely accepted dimensions of insight: awareness of illness, perceived need for treatment, and ability to re-label symptoms as pathological. Higher scores indicate greater levels of insight. The psychometric properties of the scale are excellent and it is a widely used scale in psychosis research.

### Early signs

The Early Signs Scale [40] is used at baseline and at four months to assess at risk mental states and provide a measure of clinical recovery, determined by ratings of <20 (including <10 on scales of incipient psychosis and disinhibition combined).

### Psychotic symptoms

Psychotic symptoms are measured at baseline and at four months with the Positive and Negative Syndrome Scale (PANSS; [41]). The PANSS is a 30-item semi-structured interview that consists of seven items assessing positive symptoms (e.g., hallucinations, delusions, conceptual disorganisation), seven items assessing negative symptoms (blunted affect, difficulty in abstract thinking) and 16 items assessing global psychopathology (e.g., depression, anxiety, disorientation). Items are scored between one (not present) and seven (severe). The PANSS is a widely used, valid and reliable measure of mental state.

### Duration of untreated psychosis (DUP)

At the four-month follow-up assessment, DUP is assessed with a semi-structured interview following the model of Beiser [42]. DUP is defined as the time interval between the onset of psychotic symptoms and the initiation of treatment, and calculated according to a stringent protocol adapted from criteria developed by Larsen [43].

### Premorbid functioning

Premorbid functioning is assessed at the four-month follow-up assessment with the Premorbid Adjustment Scale (PAS) [44]. The "premorbid" period is defined as the period that ends six months before there is any evidence of psychotic symptoms or first psychiatric hospital admission. The PAS measures four areas of development: (1) sociability-isolation, (2) peer relationships, (3) ability to function outside of the nuclear family, and (4) capacity to form intimate socio-sexual ties at each of four life stages: childhood (up to age 11), early adolescence (12–15 years), late adolescence (16–18 years), and adulthood (19 years and older). The PAS includes a 'general' scale that measures the highest level of functioning attained by the individual before becoming ill. For example, if an individual was aged 20 at the time of completion, but experienced psychotic symptoms at the age of 17, the adult scale would not be completed. Items are scored on a Likert-type scale of zero to six, with lower scores denoting healthier functioning and higher scores indicating greater dysfunctional adjustment.

### Pathways to care

Pathways to care is measured at four months follow-up with the Encounter form [45]. This instrument is also used to determine retrospective GP consultation and referral rates and use of the Mental Health Act from primary care records.

### Reliability of diagnoses and PANSS scores

The two field researchers attended a five day, WHO-certified training course in using the SCAN and a local two day training course in administering the PANSS to the training standard of interrater concordance between field researchers and trainers. The interrater reliability method used was descriptive, according to agreement within one rating point on the positive and negative subscales and within three rating points on the general psychopathology subscale. To achieve interrater reliability on the total PANSS scores, agreement between the field researcher and trainer had to be within the 80% range. Throughout the study, we will conduct interrater reliability maintenance checks of the SCAN and PANSS, with live interviews, to avoid drift in scoring across time.

### Sample size

The primary outcome of the study is the difference between the randomised groups on the number of young people with FEP referred to EIS during the study period, analysed on the basis of intention to treat. As the GPs, rather than their patients, are the subjects of the study, the statistical analysis and thus power calculation accounts for this.

Assuming the standard critical value for α (2 sided p = 0.05), and further that, on average, two referrals will be made in control practices in the study period, and that the variance for between practice variability is one, the study has 80% power (1-β) to detect a mean difference of 1.2 referrals between intervention and control practices, and 90% power (1-β) to detect a mean difference of 1.4 referrals.

As this study is the first randomised trial of an educational intervention in primary care which aims to influence detection and referral practice of GPs, we have no data upon which to base power calculations for the secondary outcome measures which may be considered exploratory (and which will provide relevant data for any future studies).

### Blinding

The participants and field researchers assessing patient outcomes were blinded to the identity of practices that participated in the educational intervention. Statistical analysis follows a pre-specified analysis plan.

### Statistical analysis plan

The effect of the intervention on the primary outcome will be estimated using a non linear mixed model, where the number of referrals per practice will be modelled using Poisson error, and overdispersion (extra Poissonian variability) will be accounted for by defining the practice as a random effect [46]. Analyses will be conducted using Proc Nlmixed in the SAS statistical programme, version 9 [47].

Secondary outcomes will be analysed using a mixed modelling strategy, accounting for between practice variability by defining practices as random effects. As the subject of the experiment is the practice rather than the patient, the denominator degrees of freedom for the analysis will be derived from that stratum.

### Practices recruited

A total of 78 practices out of 89 eligible practices were recruited between July 2003 and October 2003 (Figure 1). The 11 practices that declined to participate cited staffing problems and/or time pressures as the main reasons for non-participation.

### Characteristics of participating practices

The practice characteristics of participating and non-participating practices are summarised in Table 3 and suggest that the participating practices are representative and that the randomisation, after stratification by practice list size and PCT, has been effective.

**Table 3:** Practice characteristics at baseline

	Intervention Practices (*n *= 39)	Control Practices (*n *= 39)	Practices Declined (*n *= 11)
Mean practice list size (range)	4200 (924–10377)	4083 (1465–9927)	4149 (1300–9598)
Single-handed practices	17 (44%)	17 (44%)	6 (55%)
Mean No. of senior partners per practice (range)	2.35 (1–8)	2.03 (1–7)	1.81 (1–4)
Total number of GPs	80	67	20

### Attendance at education intervention and GP feedback

All 39 practices (100%) were represented by one or more GPs at the initial video-based education sessions. Feedback forms on the video were completed by 53 of the 62 GPs (85% response rate). The majority of GPs who participated in the video training agreed or strongly agreed that the video helped them to identify key information to assist their consultations (89%), highlighted useful questions to ask patients (81%), and would improve their confidence in their ability to detect FEP (66%).

### Attendance at refresher education and GP feedback

Thirty-five GPs, representing 26 practices (66.7%), attended the refresher education session and one practice (eight GPs) was visited by the first author. Feedback was obtained from 31 GPs (response rate 89%). Training was perceived as effective in raising awareness of FEP (87%) and the referral process to early intervention services (84%), and GPs reported that the training was relevant (84%), enjoyable (87%), and informative (84%).

## Discussion

The Redirect trial has been designed to evaluate the effectiveness of an educational intervention targeted at GPs on the detection of young people with FEP on referrals to EIS. The educational intervention was designed to be feasible to implement in a busy primary care setting and acceptable to GPs. Recruitment of practices into the trial itself was helped by ensuring that GP time and involvement in the study was kept to a minimum, in recognition of the workload pressures that many UK GPs currently face, particularly with the implementation of the new GMS GP contract [20]. All patients are therefore being recruited from the EIS.

The full support and co-operation of the PCTs, in particular the Heart of Birmingham PCT who enabled the study team to use the bi-monthly PCT protected learning time session to deliver the video based education, was also key to the success of implementing the intervention.

### Strengths

The practice recruitment and retention rates were excellent, ensuring the generalisability of the study findings. Seventy-eight out of 89 eligible practices were recruited, a response rate of 87.6%. To date, there has been a low drop-out rate (one practice). We used a stratified-cluster design [32] to ensure a balanced randomisation for social deprivation, which we believe may affect patient-level outcomes. We are recruiting patients directly from specialist secondary care services, and patient outcomes are based on structured interviews and self-report. The GPs positively evaluated key elements of the initial education video and refresher education, and the good attendance at both these events suggests that this educational intervention is feasible and acceptable in a primary care setting.

### Limitations

It was not possible to accurately assess whether the GPs actually used the written materials that accompanied the video (booklet and laminated 'tip' sheet) during consultations. GPs may have disseminated their copy of the written materials to control group GPs in their area and the extent of contamination between intervention and control group practices is therefore unknown.

## Conclusion

FEP is a devastating diagnosis for the individual and their family. GPs appear to be key players in the referral pathway into EIS and therefore play an important role in the early identification of FEP and potentially in reducing DUP. The Redirect trial, which will report in 2006, will provide robust information about the efficacy of an evidence-based complex educational intervention targeted at GPs on referral rates of young people with FEP to EIS.

## Competing interests

The author(s) declare that they have no competing interests.

## Authors' contributions

HL, SW, and MB participated in the design of the study and wrote the protocol. LT contributed to the further development of the study design and protocol. NF is involved in the analysis and interpretation of the data. LT and HL participated in the drafting of the manuscript. All authors have contributed to and approved the final manuscript.

## Pre-publication history

The pre-publication history for this paper can be accessed here:


